# Enhancing Digital Capacity of Vulnerable Older Adults in Hong Kong

**DOI:** 10.1093/geroni/igaf122.863

**Published:** 2025-12-31

**Authors:** Xue Bai, Youjuan Zhang, Shuai Zhou

**Affiliations:** The Hong Kong Polytechnic University, Hong Kong, China (People’s Republic); The Hong Kong Polytechnic University, Hong Kong, China (People’s Republic); The Hong Kong Polytechnic University, Hong Kong, China (People’s Republic)

## Abstract

The global digital revolution has reshaped society, yet older adults, particularly vulnerable ones, struggle to adapt, lagging behind due to limited access and skills. In Hong Kong, where the aging population is projected to rise from 1.45 million in 2021 to 2.74 million by 2046 (20.5% to 36.0%), and despite a leading ICT development index, a triple digital divide—access, capability, and outcomes—persists. Research shows older adults face secondary and tertiary divides, with inadequate digital literacy hampering technology use, a gap widened during the pandemic when digital reliance surged unevenly. Strengthening their digital capacity is vital to reduce loneliness, enhance memory, and boost well-being. The “Evergreen Digital Fuel Station—Fun Fun Needs You” project, funded by the Social Innovation and Entrepreneurship Development Fund and hosted by The Hong Kong Polytechnic University’s Centre for Gerontology and Family Studies, addresses this through a community-based approach. This initiative promotes intergenerational inclusion via diverse training, including three-tiered, six-module courses, consultation points, and post-training activities like service and documentary production. Integrating the PERMA model, it fosters positive engagement, peer support, and lifelong learning. Evaluations reveal improved digital literacy, reduced anxiety, and enhanced mental health among participants—predominantly female retirees with moderate education—especially in the positive education group. Qualitative feedback highlights increased autonomy, shifted stereotypes, and stronger intergenerational bonds. This scalable model narrows the digital divide, empowers older adults, and fosters a cohesive, age-friendly society.

